# Does Birth Trigger Cell Death in the Developing Brain?

**DOI:** 10.1523/ENEURO.0517-19.2020

**Published:** 2020-02-10

**Authors:** Alexandra Castillo-Ruiz, Taylor A. Hite, Dina W. Yakout, T. John Rosen, Nancy G. Forger

**Affiliations:** 1 Neuroscience Institute, Georgia State University, Atlanta, GA 30302; 2 Department of Mathematics and Statistics, Georgia State University, Atlanta, GA 30302

**Keywords:** apoptosis, birth, birth timing, cell death, cesarean, parturition

## Abstract

Developmental cell death eliminates half of the neurons initially generated in the mammalian brain, and occurs perinatally in many species. It is possible that the timing of neuronal cell death is developmentally programmed, and only coincidentally associated with birth. Alternatively, birth may play a role in shaping cell death. To test these competing hypotheses, we experimentally advanced or delayed birth by 1 d in mice (within the normal range of gestation for the species) and examined effects on the temporal pattern and magnitude (amount) of neuronal cell death, using immunohistochemical detection of activated caspase-3 as a cell death marker. In order to detect effects of subtle changes in birth timing, we focused on brain areas that exhibit sharp postnatal peaks in cell death. We find that advancing birth advances peak cell death, supporting the hypothesis that birth triggers cell death. However, a delay of birth does not delay cell death. Thus, birth can advance cell death, but if postponed, a developmental program governs. Advancing or delaying birth also caused region-specific changes in the overall magnitude of cell death. Our findings shed light on the long-standing question of what controls the timing and magnitude of developmental neuronal cell death, and position birth as an orchestrator of brain development. Because humans across the world now routinely alter birth timing, these findings may have implications for current obstetric practices.

## Visual Abstract

**Figure:**
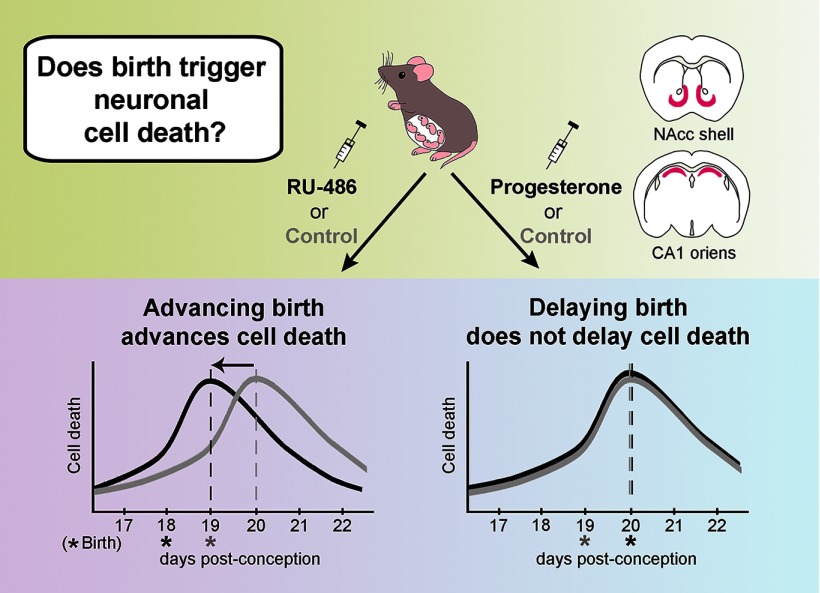

## Significance Statement

The importance of neuronal cell death for brain development has been recognized for decades, but it is unknown what regulates its timing, or accounts for differences in the amount of cell death between brain regions. In many species, including mice, developmental cell death occurs perinatally. We find that advancing birth by 1 d in mice advances patterns of cell death, but does not advance overall forebrain (FB) growth. Because humans across the world routinely alter birth timing, usually to advance birth, our findings may have implications for current obstetric practices. Birth timing also affects the magnitude of cell death in a region-specific manner, suggesting that birth has important, previously unrecognized, effects on brain development.

## Introduction

Developmental cell death is an important feature of brain development in all vertebrates. In mammals, approximately 50% of the neurons initially generated are eliminated during a discrete perinatal period (Oppenheim, 1991; Dekkers et al., 2013). Developmental neuronal cell death occurs by apoptosis, and culminates in the activation of caspases, including caspase-3, which causes cellular demise through the cleavage of key intracellular proteins (Porter and Jänicke, 1999; Hengartner, 2000). Although the importance of this process has been recognized for over 50 years, many basic questions still remain. It is unknown, for example, what initiates or terminates the cell death period. Specifically, does the timing of neuronal cell death reflect intrinsic factors (i.e., an autonomous developmental program), or do extrinsic factors play a role? Also, what accounts for the large (>10-fold; Ahern et al., 2013) regional differences in the magnitude of cell death? The tight association of cell death with birth in mice provides an opportunity to address these questions.

A cell death “atlas” of the mouse brain was recently compiled, using immunohistochemistry for activated caspase-3 (AC3) to quantify apoptosis in over 20 forebrain (FB) regions prenatally and postnatally (Ahern et al., 2013; Mosley et al., 2017). Although AC3 has been reported in the absence of apoptosis, the large majority of cells positive for AC3 in the perinatal rodent brain co-label with other cell death markers, and are neurons (Srinivasan et al., 1998; Zuloaga et al., 2011; Ahern et al., 2013). Results of the atlas study show that the cell death period is largely compressed into the first postnatal week in mice, with the highest number of dying cells seen during the first 3 d after birth in many brain regions (Ahern et al., 2013; Mosley et al., 2017). One possible explanation for these findings is that extrinsic factors associated with labor, parturition, and/or the transition to *ex utero* life may determine cell death timing. If so, then advancing or delaying birth would be expected to advance or delay cell death, respectively. Alternatively, if the timing of cell death is based on an autonomous developmental program, then patterns would depend on postconceptional age, independent of birth timing.

To distinguish between these hypotheses, we manipulated birth timing in C57BL/6 mice. Most births in this strain occur at 19 d postconception (dpc; Murray et al., 2010), but a range of 18–20 d is considered normal (Donahue, 2012). We randomly assigned timed-pregnant dams to give birth early (18 dpc), on-time (19 dpc), or late (20 dpc) within this range. Parturition in mice is normally triggered when the corpus luteum regresses, leading to an abrupt fall in progesterone (Ratajczak and Muglia, 2008; Mitchell and Taggart, 2009). Although there are several ways to control parturition timing (e.g., ovariectomy, gene knock-outs), the approach we took here comes closest to replicating how short and long gestations naturally vary from each other, i.e., we mimicked the hormonal profiles of an “advanced” or “delayed” parturition by injecting a progesterone receptor antagonist to advance birth, or by clamping progesterone levels to delay birth (Dudley et al., 1996; Hashimoto et al., 2010).

In order to be able to discern effects of a subtle change in gestation length (i.e., 1 d), we required brain regions with discrete neonatal peaks in cell death, and large changes in apoptosis from one day to the next. The cell death atlas study described above allowed us to identify two brain regions fulfilling these criteria: the CA1 oriens layer of the hippocampus (CA1or) and the shell of the nucleus accumbens (NAcc). Both of these regions show a sharp increase in cell death in the 24 h after birth, followed by a rapid decline over the next 2 d (Ahern et al., 2013; Mosley et al., 2017).

We reasoned that if neuronal cell death is independent of birth, then patterns will be determined by postconceptional age, regardless of when pups are delivered. Alternatively, if birth plays an important role in triggering cell death, patterns will be determined by days postnatal, independent of days postconception. We find that advancing birth advances cell death patterns, suggesting that an early birth can trigger cell death. In contrast, cell death depends on postconceptional age if birth is delayed. We also find that birth timing influences the magnitude of cell death in a brain region-specific manner.

## Materials and Methods

### Animals and timed pregnancies

Adult female and male C57BL/6 mice were obtained from our breeding program or purchased from The Jackson Laboratory. Mice were maintained on a 12/12 h light/dark cycle with *ad libitum* access to food and water. All animal procedures were performed in accordance with the Georgia State University animal care committee’s regulations. We established timed-pregnancies by pairing animals within an hour of lights off and removing males 1–2 h after lights on. The morning after pairing was considered embryonic day 0 (0 dpc) and cages were checked four to six times a day for births.

### Advancing birth

To advance birth, we injected pregnant dams at 17 dpc with RU-486 (150 μg in 0.1-ml sesame oil; Sigma-Aldrich; catalog #M8046; *N *=* *7), as previously described (Dudley et al., 1996; Toda et al., 2013). RU-486 is an antagonist of both progesterone and glucocorticoid receptors (Baulieu, 1989). When administered to the mother, it is detected in the fetal circulation (albeit at much lower levels) and does not affect estradiol, glucocorticoid, or progesterone levels in the mother or the fetus (Wolf et al., 1988; Hill et al., 1990). Injections were administered five to 10 min before lights off, and controls received vehicle injections (*N *=* *9) on the same schedule. All RU-486-treated dams gave birth on 18 dpc, within 18 h of treatment. Pups of both sexes were weighed, euthanized, and their brains collected on postnatal day (P)0 (the day of birth), P1, and P2 (between 9 and 11 h after lights-on each day). During the course of this study, one unmanipulated timed-pregnant dam from our breeding colony and three vehicle-treated dams also gave birth on 18 dpc. As there were no differences in cell death between the offspring delivered on 18 dpc “spontaneously” or after RU-486 treatment (Extended Data Fig. 1-1), data from all advanced births were pooled in the analyses below.

**Figure 1.**
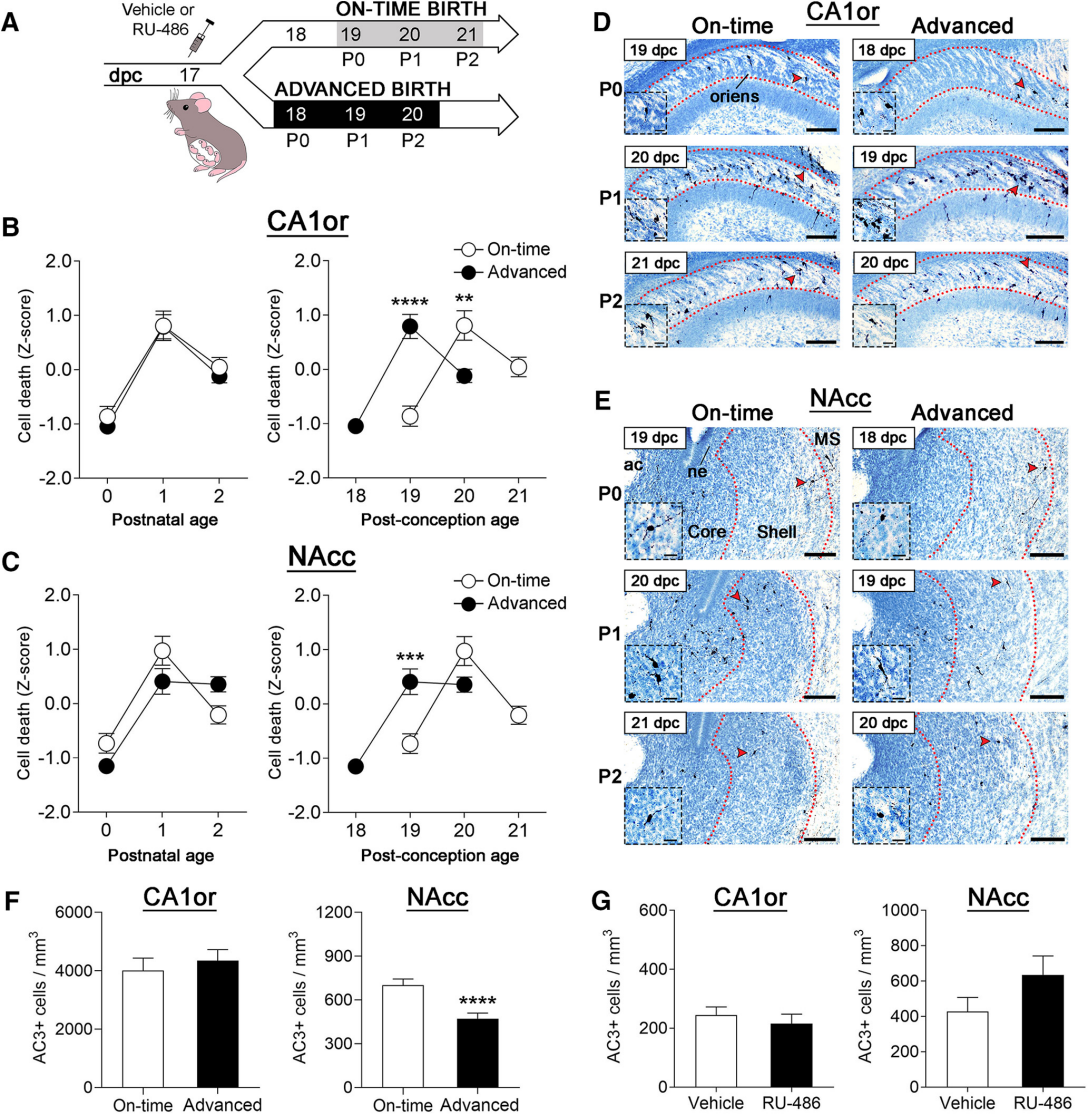
Advancing birth by 1 d advances cell death. ***A***, Timeline of experimental procedures. ***B***, ***C***, Cell death was very similar between the on-time and advanced groups when compared by postnatal age (left), but not by postconception age (right) in both the CA1or and NAcc. Asterisks indicate significant differences by Bonferroni *post hoc* tests: **p < 0.01, ***p < 0.001, ****p < 0.0001. In addition, cell death did not differ between pups with spontaneous advanced birth versus those with advanced birth after RU-486 treatment of the dam (Extended Data Fig. 1-1). ***D***, ***E***, Photomicrographs of AC3-labeled tissue (counterstained with thionin) in representative sections from the on-time and advanced birth groups in the CA1or and NAcc. Arrowheads point to cells shown at higher magnification in the insets. Peak cell death occurred at P1, despite the fact that this was 20 dpc for on-time births and 19 dpc for advanced births; ac, anterior commissure; MS, medial septum; ne, neuroepithelium. Scale bars = 100 μm (main photograph) and 20 μm (insets). ***F***, Advancing birth by a day did not change the magnitude of cell death over the first three postnatal days in the CA1or, but decreased cell death in the NAcc; ***p = 0.0001. ***G***, RU-486 did not alter cell death in fetuses 3 h after treatment of the dam in either the CA1or or NAcc. Means ± SEM are plotted for all figures except for ***F***, which shows estimated marginal means ± SEM.

10.1523/ENEURO.0517-19.2020.f1-1 Extended Data Figure 1-1 Cell death patterns are similar between mice with spontaneous advanced birth and those with advanced birth after RU-486 treatment. AC3 cell density in the CA1or (***A***) and NAcc (***B***) are depicted for mice spontaneously delivered at 18 dpc (dotted lines) versus those delivered at 18 dpc after RU-486 treatment of the dam (hashed lines). In both cases, cell death is very low on P0 and increases significantly on P1, with no significant differences between groups; n.s., non-significant; *N *=* *8–12 mice per group. Means ± SEM are plotted. Download Figure 1-1, TIF file.

### Delaying birth

To delay birth, we injected dams with progesterone (0.5–1 mg in 0.1- to 0.2-ml vehicle; Sigma Aldrich; catalog #P3972; *N *=* *23), as per (Hashimoto et al., 2010) on 17 and 18 dpc, 5 h after lights on. Controls received the vehicle (2% ethanol in peanut or sesame oil; *N *=* *5). Most (*N* = 12; 52%) of the progesterone-treated dams gave birth on 20 dpc, and the offspring of eight of these pregnancies were used in the main experiment. Two treated dams (9%) gave birth on 19 dpc and nine (39%) gave birth on 21 dpc; a subset of the pups born on 21 dpc (from five different litters) were also examined, as described below. Births tended to occur within 6 h after lights on, and pups of both sexes were weighed, euthanized, and their brains collected on P0, P1, and P2 (between 9 and 11 h after lights on each day).

### Effects of RU-486 and progesterone on cell death *in utero*

To test for effects of RU-486 and progesterone on neuronal cell death in the offspring *in utero*, timed-pregnant dams were treated as above. Three hours after the last injection, dams were anesthetized with 2% CO_2_ and rapidly decapitated. The fetuses were removed from the uterine horns, and their brains collected and processed for the detection of AC3. We also assessed progesterone levels in the offspring of progesterone- and vehicle-treated dams by ELISA. Trunk blood was collected and plasma from two to six embryos was pooled to generate each sample. Samples were analyzed in duplicate at the University of Virginia Center for Research in Reproduction Ligand Core Laboratory according to the manufacturer’s recommendations (Immuno-Biological Laboratories Inc.).

### Immunohistochemistry

Brains were immersion fixed in 5% acrolein for 24 h, followed by 30% sucrose for at least 48 h, and then transferred to cryoprotectant solution at −20°C until sectioning. Brains were coronally frozen-sectioned into four, 40-μm series, and two series were used for the immunohistochemical detection of dying cells, as previously (Ahern et al., 2013; Mosley et al., 2017; Castillo-Ruiz et al., 2018a,b). Briefly, epitope retrieval and unreacted aldehyde blockade was performed with 30-min incubations in 0.05 M sodium citrate and 0.1 M glycine, respectively. Tissue was then incubated in a blocking solution (20% normal goat serum and 1% hydrogen peroxide) for 30 min, followed by an overnight incubation in AC3 primary antibody (Cell Signaling; catalog #9661, RRID:AB_2341188; 1:20,000 for newborns and 1:10,000 for fetuses). On the following day, the tissue was incubated with goat anti-rabbit secondary antibody (Vector Laboratories; catalog #BA-1000, RRID:AB_2313606; 1:500) for 1 h, and then with avidin and biotin (Vector Laboratories; catalog #PK-6100; 1:500) for 1 h. The tissue was reacted for 30 min with 3,3’-diaminobenzidine tetrahydrochloride, nickel sulfate, and hydrogen peroxide. Tissue was mounted onto gelatin-coated slides, counterstained with thionin, and coverslipped.

### Quantification of AC3 labeling

For analysis of cell death in newborn mice, slides were scanned using a Hamamatsu Nanozoomer (Hamamatsu Photonics K.K.) and AC3+ cell quantification was performed using Aperio Image Scope (Leica Biosystems Inc). For the smaller experiments in which cell death was examined in fetuses, AC3 immunostaining was quantified live on a microscope using Stereo Investigator software (MBF Biosciences). In both cases, we drew contours around the areas of interest and AC3+ cells within each contour were quantified as previously described (Ahern et al., 2013; Mosley et al., 2017; Castillo-Ruiz et al., 2018a,b). Because AC3 cells are relatively sparse, all positive cells could be counted and random sampling was not required. Cells were counted only when the cell body was clearly visible within the section. The sum of AC3+ cell counts across all sections was divided by total area sampled, and then multiplied by section thickness to obtain the density of AC3+ cells per mm^3^ for each animal. Investigators blind to experimental conditions performed all analyses.

### Brain regions and total FB volume

For the CA1or, our sampling spanned the dorsal hippocampus. We analyzed sections starting where the dentate gyrus makes its rostral-most appearance (Paxinos et al., 2007, their Fig. 65 of the developing mouse brain atlas), and ending when the lateral extent of the hippocampus starts to tip ventrally (Paxinos et al., 2007, their Fig. 70 of the developing mouse brain atlas) as described in (Mosley et al., 2017); in general, this included four alternate sections per animal. For the shell of the NAcc, we included sections starting at the point at which the corpus callosum joins at midline (Paxinos et al., 2007, their Fig. 54 of the developing mouse brain atlas) to the point where the anterior commissure starts elongating horizontally (Paxinos et al., 2007, their Fig. 58 of the developing mouse brain atlas); in general, this included six alternate sections per animal. We estimated overall brain size by outlining the left side of the entire FB from the point where the medial border of the anterior commissure is located ventral to the tip of the lateral ventricle to the rostral most appearance of the dorsomedial nucleus of the hypothalamus (Paxinos et al., 2007, their Figs. 57–70 of the developing mouse brain atlas) as described previously (Castillo-Ruiz et al., 2018a).

### Statistical analyses

Statistical analyses were performed using IBM SPSS Statistics v.25 (IBM Corp.) and GraphPad Prism v.8 (GraphPad Software, Inc.). We first performed three-way ANOVAs (group × postnatal age × sex) for all dependent measures (i.e., cell death, FB size, and body weight). No significant effects of sex were identified for any measure; the data from males and females therefore were pooled and two-way ANOVAs (group × postnatal age, OR group × postconceptional age) were used in the statistical analyses. Because changing the timing of birth could have influenced the pattern and/or the magnitude of cell death, we evaluated these possibilities separately. Cell death pattern matching between treatment groups was performed by converting AC3 data to Z-scores, with mean and standard error calculated per treatment group; this eliminated group differences due only to magnitude. The magnitude of cell death was then compared between treatment groups using the raw scores. Main effects and significant interactions were followed by Bonferroni tests. Two-tailed independent *t* tests were used to test for the acute effects of RU-486 or progesterone treatment in fetuses.

## Results

### Advancing birth by 1 d advances cell death

Neuronal cell death was compared on P0, P1, and P2 in mice born “on time” or 1 d early (Fig. 1*A*; *N *=* *12–20 mice per group). This corresponded to 19, 20, and 21 dpc for all on-time births, and 18, 19, and 20 dpc for “advanced births” (Fig. 1*A*). Birth timing could affect the temporal pattern of cell death, the overall magnitude (amount), or both. To test for effects on the pattern, independent of changes in magnitude, AC3 cell densities were transformed to Z-scores (within treatment group) and double-plotted to contrast the competing hypotheses, i.e., with either postnatal age or postconception age on the *x*-axis (Fig. 1*B*,*C*). If birth triggers cell death, then cell death patterns would be similar between groups when plotted as a function of postnatal age. On the other hand, if cell death is developmentally programmed, then the pattern of cell death should depend on postconceptional age, regardless of day of birth.

The prediction that birth timing determines cell death timing was supported. In the CA1or and NAcc, a prominent peak in cell death was previously reported 1 d after birth (P1 = 20 dpc; Mosley et al., 2017). Here, peak cell death again occurred 1 d after birth (P1) in both brain regions and for both groups, despite the fact that this was 19 dpc for the advanced-birth group and 20 dpc for the on-time birth group (Fig. 1*B–E*). In agreement, two-way ANOVA found no differences in cell death between birth groups when analyzed with respect to postnatal age in either the CA1or or NAcc. In contrast, when the data were analyzed as a function of postconception age, there was a significant interaction between days postconception and group in both regions (CA1or: *F*_(1,59)_ = 38.14, *p* < 0.0001; NAcc: *F*_(1,59)_ = 16.45, *p* = 0.0001), reflecting the fact that peak cell death occurred on different days postconception for the groups (Fig. 1*B*,*C*). [Note: the ANOVAs for postnatal age included all time points (P0, P1, P2) and those for postconception age included only timepoints for which both groups were represented (19 and 20 dpc).]

### Advancing birth decreases overall cell death in the NAcc

Next, to determine whether birth timing affected the magnitude of cell death independent of the temporal pattern, we calculated the main effect of group on cell death across all three postnatal days, this time using the raw number of dying cells (*N *=* *38–52 mice per group). Advancing birth did not alter the magnitude of cell death in the CA1or (Fig. 1*F*). However, overall cell death in the advanced birth group was reduced by approximately 33% in the NAcc compared with controls (*F*_(1,84)_ = 16.09, *p* = 0.0001; Fig. 1*F*).

### RU-486 does not alter cell death independent of effects on birth timing

The fact that cell death patterns in RU-486-treated offspring did not differ from those of neonates spontaneously delivered on 18 dpc (Materials and Methods; Extended Data Fig. 1-1) supports the conclusion that changes in birth timing, rather than some other effect of RU-486, caused an advance in cell death. To further assess whether RU-486 treatment of dams alters neuronal cell death in their offspring independent of birth, a new cohort of timed-pregnant dams was treated with vehicle or RU-486, as above (Fig. 1*A*). When administered to the mother, RU-486 is found in the fetal circulation within a few hours (Wolf et al., 1988; Hill et al., 1990). We therefore collected the brains of offspring 3 h after injection (*N *=* *12–17 mice per group), while all offspring were still *in utero,* and processed them for the detection of AC3. We found no effect of RU-486 treatment on cell death *in utero* in either the CA1or or NAcc (Fig. 1*G*).

### Delaying birth by 1 d does not delay cell death

**Figure 2.**
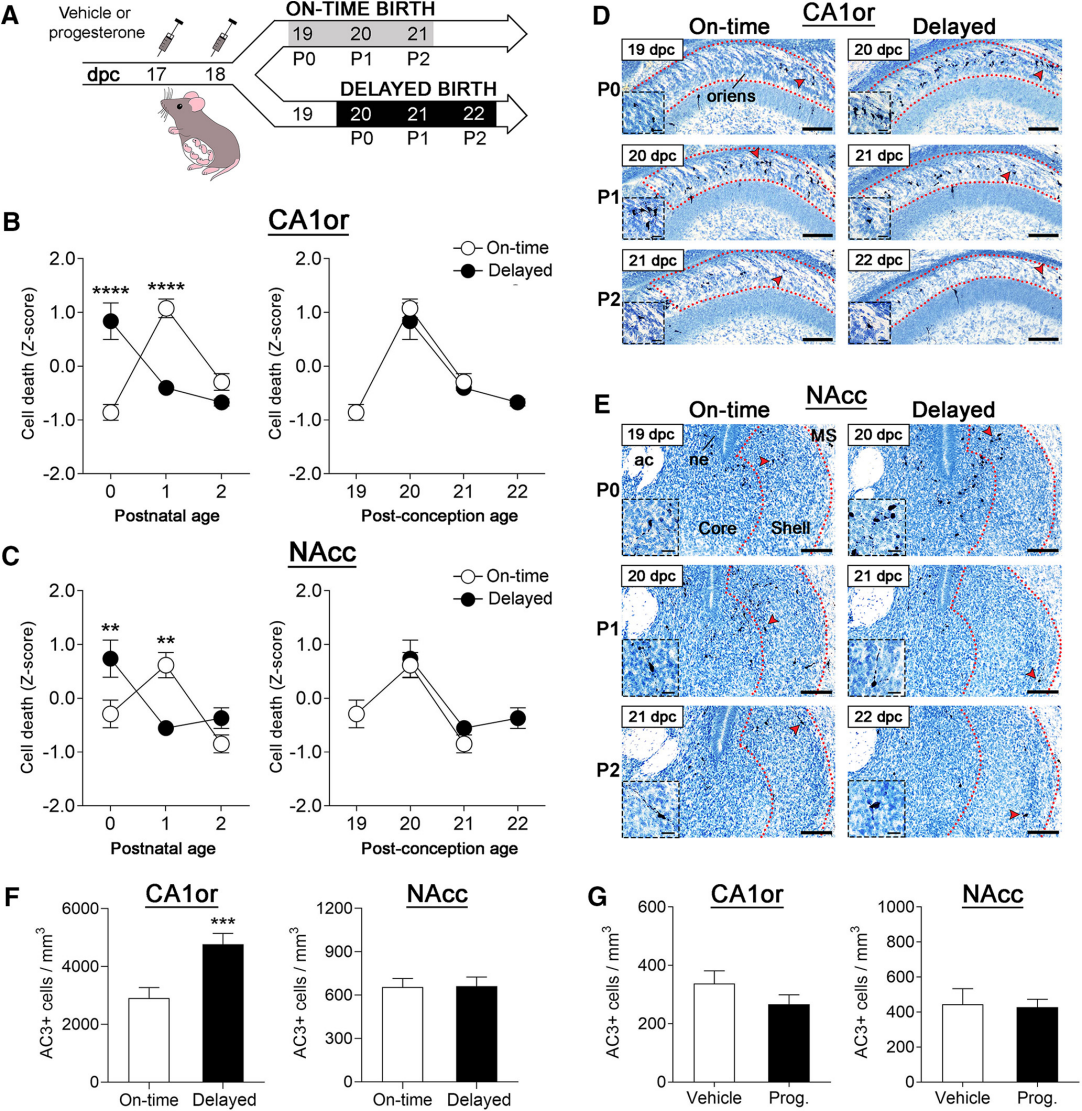
Delaying birth by a day does not delay cell death. ***A***, Timeline of experimental procedures. ***B***, ***C***, Patterns of cell death in on-time versus delayed birth groups best match when plotted as a function of postconception age (right), rather than postnatal age (left) in both the CA1or and NAcc. Asterisks indicate significant differences on each day by Bonferroni tests: **p < 0.01, ****p < 0.0001. ***D***, ***E***, Photomicrographs of AC3-labeled tissue (counterstained with thionin) in representative sections of mice from the on-time and delayed birth groups in the CA1or and NAcc. Arrowheads point to cells shown at higher magnification in the insets. Peak cell death occurred at 20 dpc, despite the fact that this was P1 for the on-time birth group and P0 for the delayed-birth group; ac, anterior commissure; MS, medial septum; ne, neuroepithelium. Scale bars = 100 μm (main photograph) and 20 μm (insets). ***F***, Delaying birth by a day increased cell death magnitude overall in the CA1or but did not affect the magnitude of cell death in the NAcc; ***p = 0.0007. ***G***, Progesterone treatment of the dams increased progesterone levels in fetuses (as described in Results) but did not affect cell death in either brain region. Means ± SEM are plotted for all figures except for ***F***, which shows estimated marginal means ± SEM.

If advancing birth advances cell death, a delay in birth may delay cell death. Alternatively, endogenous signals might trigger cell death when birth is delayed. To test this, we treated timed-pregnant dams with progesterone on 17 and 18 dpc to delay parturition (Hashimoto et al., 2010) by a day; i.e., birth occurred on 20 dpc (“delayed birth” group). Their offspring were compared with those of vehicle controls which gave birth at 19 dpc (on-time birth). The brains of offspring were again examined on P0, P1, and P2 (*N *=* *9–12 mice per group), which in this case corresponded to 19, 20, and 21 dpc for the on-time group and 20, 21, and 22 dpc for the delayed birth group (Fig. 2*A*).

**Figure 3.**
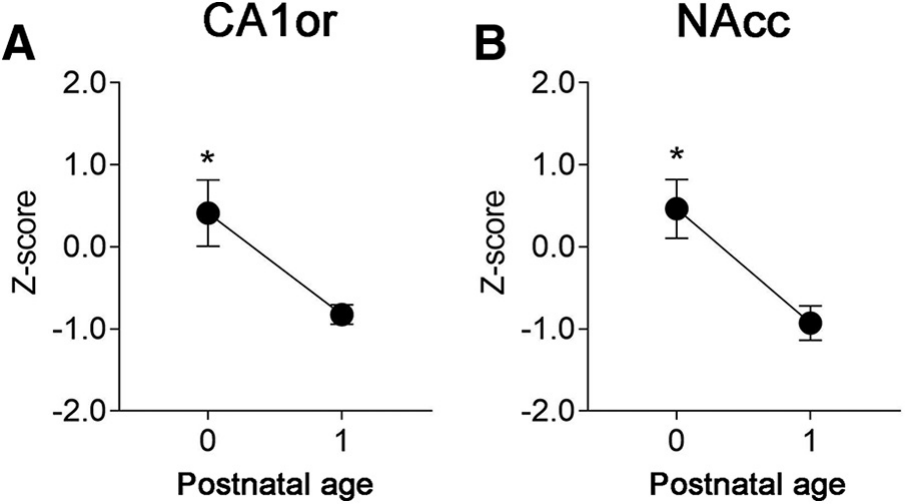
Cell death declines between P0 and P1 in pups born 2 d late. Cell death in the CA1or (***A***) and NAcc (***B***) was higher on P0 than on P1 (*p *=* *0.04 and *p *=* *0.02, respectively) in pups born 2 d late (on 21 dpc) to dams treated with progesterone on days 17 and 18 postconception. This outcome is in contrast to that seen for mice born on-time (where cell death in the CA1 and NAcc is low on P0 and peaks on P1; Figs. 1, 2*B*,*C*) but is expected if a developmental program governs cell death when birth occurs late; *N *=* *3–6 mice per group. Means ± SEM are plotted, *p < 0.05.

Contrary to what was seen with advancing birth, postconceptional age, rather than postnatal age, best predicted cell death patterns when birth was delayed. For both the CA1or and NAcc, the peak of cell death occurred at 20 dpc, despite the fact that this was P1 for the on-time group and P0 for the delayed group (Fig. 2*B–E*). Two-way ANOVAs (group × postnatal age) confirmed a significant interaction in both regions (CA1or: *F*_(2,58)_ = 35.42, *p* < 0.0001; NAcc: *F*_(2,60)_ = 11.43, *p* < 0.0001), due to the fact that peak cell death occurred on different days postnatal (Fig. 2*B*,*C*). In contrast, when the data were analyzed as a function of postconceptional age, the two-way ANOVA found no differences between the groups and no group × age interaction (Fig. 2*B*,*C*). Further supporting these findings, we examined cell death in the offspring of five progesterone-treated dams that gave birth on 21 dpc (i.e., 2 d after expected delivery). Again, cell death was higher on P0 than on P1 in the CA1or and NAcc (Fig. 3), which is predicted if cell death is driven by a developmental program.

### Delaying birth increases overall cell death in the CA1or

To determine whether a delay in birth altered overall cell death magnitude, we calculated the main effect of group on AC3 cell number across all three postnatal ages. A delay in birth increased cell death magnitude overall by approximately 64% in the CA1or (*F*_(1,58)_ = 12.72, *p* = 0.0007), but had no effect in the NAcc (Fig. 2*F*). Thus, delaying birth does not delay the timing (i.e., temporal pattern) of developmental cell death, but may cause region-specific differences in magnitude.

### Progesterone treatment does not acutely alter cell death

To determine whether progesterone treatment of dams alters cell death of fetuses *in utero*, a separate cohort of timed-pregnant dams was treated with progesterone or vehicle on 17 and 18 dpc (as above; Fig. 2*A*). The trunk blood and brains of offspring were collected 3 h after the last injection (27 h after the first injection) to measure circulating progesterone (*N *=* *7–9 pooled samples per group) and AC3-cell number (*N *=* *12–26 mice per group), respectively. As expected, progesterone levels were significantly increased in the offspring of treated dams (3.67 vs 29.19 ng/ml for vehicle and progesterone-treated samples; *p *=* *0.0002). However, there was no effect on cell death in either the CA1or or NAcc (Fig. 2*G*).

### Overall brain growth is determined by days postconception

**Figure 4.**
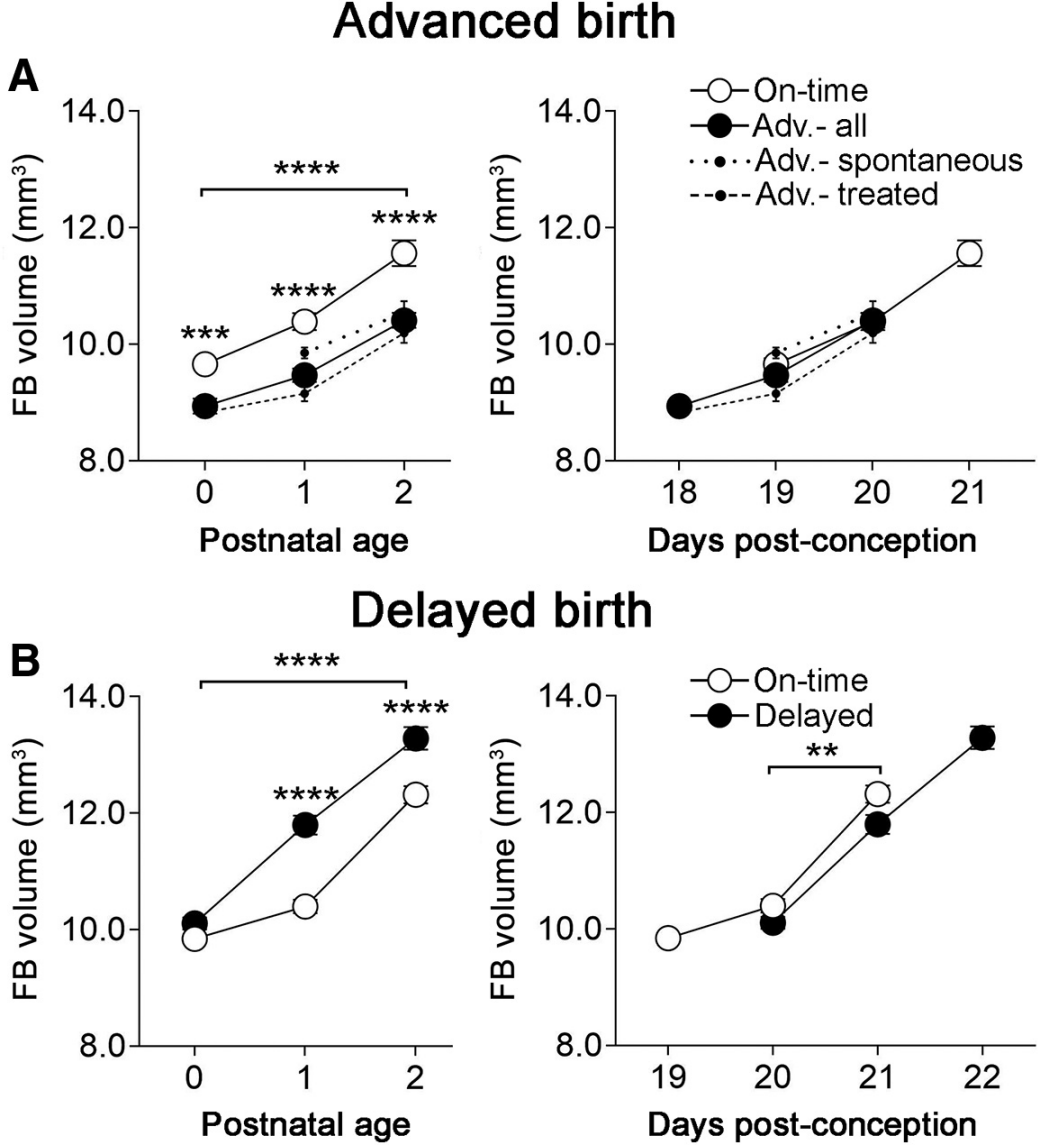
Overall brain growth is determined by postconceptional age. FB growth was best predicted by postconception age (right), rather than postnatal age (left) whether birth was advanced (***A***) or delayed (***B***). Because mice with spontaneous advanced births differed from mice with advanced birth after RU-486 treatment on this measure, the data for those groups are plotted separately (dotted and hashed lines, respectively), as well as combined (solid lines). However, the comparison between on-time and advanced groups remained significant whether considering spontaneous only, treated only, or all advanced births (*p* < 0.0001 in each case). Asterisks indicate significant differences on each day by Bonferroni *post hoc* tests. Brackets indicate main effect of group. **p < 0.01, ***p =* *0.001, ****p <* *0.0001. Means ± SEM are plotted.

It was possible that the advance of cell death patterns following an early birth was an indirect effect of an overall acceleration in brain development. To address this, we calculated total FB volume for all animals in the advance- and delay-birth experiments (*N *=* *9–20 mice per group). Interestingly, FB volume depended on postconceptional age regardless of whether birth was advanced or delayed. When analyzed with respect to postnatal age, there were highly significant group differences in FB size between on-time and advanced births (*F*_(1,81)_ = 69.06, *p* < 0.0001; Fig. 4*A*, left), as well as between on-time and delayed births (*F*_(1,60)_ = 56.91, *p* < 0.0001; Fig. 4*B*, left). By contrast, when analyzed by postconceptional age, there was no difference in FB size between advanced birth and on-time groups (Fig. 4*A*, right). There was a subtle effect of group when birth was delayed (*F*_(1,41)_ = 9.68, *p* = 0.0034; Fig. 4*B*, right), although the difference was not significant for any single age in the *post hoc* tests.

**Figure 5.**
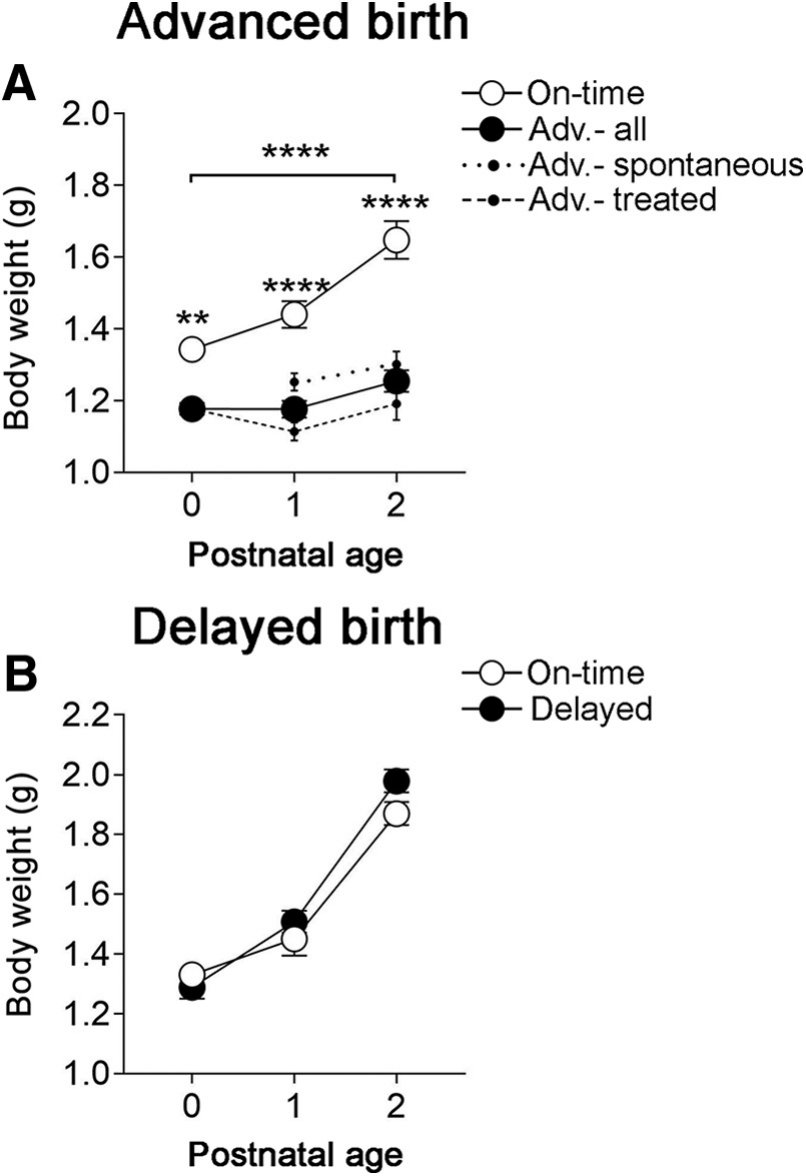
Effects of an advanced or delayed birth on overall body growth. ***A***, When birth was advanced (filled circles), body growth lagged behind that of pups delivered on-time (open circles) for all postnatal ages (*F*_(1,83)_ = 98.16, ****p < 0.0001 for main effect of group; brackets). Because mice with spontaneous advanced births (dotted lines) differed from mice with advanced birth after RU-486 treatment (hashed lines) on this measure, the data for those groups are plotted separately. However, the comparison between on-time and advanced groups remains significant whether considering spontaneous only, treated only, or all advanced births (solid line, *p* < 0.0001 in each case). Asterisks indicate significant differences on each day by Bonferroni tests: **p < 0.01, ****p < 0.0001. ***B***, In contrast, body weight did not differ following an on-time versus delayed delivery (*F*_(1,60)_ = 1.62, *p *=* *0.21); *N* = 9–20 mice per group. Means ± SEM are plotted.

Thus, a generalized advance of brain development is unlikely to account for our cell death results (above) and, as a result, cell death decouples from overall brain development in animals experiencing advanced birth. The temporal pattern of cell death was also dissociated from overall body growth. Body weight lagged behind that of controls at all postnatal ages in the advanced-birth group, and was predicted by postnatal age, rather than postconceptional age, following a delayed birth (Fig. 5).

## Discussion

Although neuronal cell death clusters around the time of birth in many species, a causal relationship between birth and cell death has not been probed. Indeed, it is tricky to do so: manipulations of birth timing must be made within a narrow time window in order to avoid possible confounds associated with prematurity and postmaturity, and this, in turn, requires the identification of brain regions with striking day to day changes in cell death. Using quantitative cell death data from a previous study to identify appropriate brain regions and an approach that mimics natural hormonal variations around the time of delivery in mice, we found that advancing birth advances patterns of developmental neuronal cell death. However, if birth is delayed, cell death occurs on time. These findings therefore support both of our original hypotheses: environmental signals can determine cell death timing, but in the absence of those signals a developmental program governs.

One limitation of the current study is that we focused our analyses on two brain regions with relatively sharp postnatal peaks in apoptosis. On the one hand, this was a strategic choice that allowed us to test our hypotheses. However, it is possible that our results are not representative of all brain regions if, for example, we unintentionally selected for regions sensitive to birth timing. In fact, we attempted to quantify cell death in several other regions in the material at hand, but results were inconclusive, as it is difficult to clearly discern the effect of a 1-d change in gestation length in brain regions that do not exhibit a sharp peak in cell death. It is possible that with a very large number of animals per group, or in longer-lived species with more protracted cell death periods, this could eventually be addressed. Nonetheless, this study provides proof-of-concept that extrinsic factors such as birth can drive cell death patterns and, in so doing, enables us to address a long-standing question about the timing of developmental neuronal cell death.

Another potential concern is that our treatments themselves may have influenced cell death. It is reassuring that there was no difference in cell death in either the CA1 or NAcc in animals born early spontaneously versus after RU-486 treatment. We also did not find evidence for an acute change in cell death in either brain region after RU-486 or progesterone treatment of the dams, and progesterone was clearly elevated in fetuses at the timepoint sampled. However, we examined only a single, relatively early timepoint in each case. Although steroid hormones can affect cell death quite rapidly (Mann et al., 2000; Estrada et al., 2006; Waters and Simerly, 2009; Morrissy et al., 2010), we cannot rule out the possibility that we missed effects that may have occurred at a time not sampled here.

“Birth” is a complex stimulus, accompanied by marked hormonal changes, the mechanical stimuli associated with labor and delivery, and many physiological changes required for the fetus to survive *ex utero*. Which of these factors is responsible for advancing cell death patterns following an early birth remains to be determined. In addition to the obligatory drop in progesterone, a vaginal birth triggers an “adaptive stress response” which leads to marked increases in vasopressin, glucocorticoids, and catecholamines in the offspring (for review, see Lagercrantz and Slotkin, 1986; Evers and Wellmann, 2016). Neuronal cell death can be curbed or promoted by each of these hormones (Noh et al., 1999; Mulholland et al., 2005; Noguchi et al., 2008; Chen and Aguilera, 2010; Zuloaga et al., 2011), and receptors for them are widespread across the perinatal brain (Yi et al., 1994; Happe et al., 2004; Quadros et al., 2007, 2008; Hammock, 2015). Thus, it is possible that hormonal factors associated with labor or parturition may play a role in triggering cell death when birth is advanced. In addition, birth is an inflammatory event, marked by a surge in proinflammatory cytokines in amniotic fluid and a peripheral immune response in the newborn (Osman et al., 2003; Golightly et al., 2011; Walker et al., 2011; Kaiser et al., 2014; Kingsbury and Bilbo, 2019), which could also influence neuronal cell death. It may be possible in future studies to manipulate individual hormones or immune signaling molecules to test their roles in the control of cell death.

To our knowledge, only one other study has examined how a change in birth timing affects a well-charted neurodevelopmental event. Toda and colleagues found that when birth was advanced by 1 d in mice, the initiation of barrel formation in somatosensory cortex was accelerated (Toda et al., 2013; delayed birth was not examined in this study). This effect was related to the fact that birth triggers a decrease in extracellular serotonin, which permits barrel formation (Toda et al., 2013). Together with the present results, this suggests that an advanced birth may organize multiple events across the developing brain. However, effects are specific because an early birth does not accelerate overall brain development. In fact, global measures such as total FB volume in the current study, cortical thickness in the Toda et al. study (Toda et al., 2013), and brain weight in pigs (Holme Nielsen et al., 2018), all depend on postconceptional and not postnatal age.

In contrast to the effects of advancing birth, a delay of birth did not delay cell death patterns, supporting the existence of a developmental program. In a related study, Southwell et al. (2012) transplanted cortical interneurons from 13.5- or 14.5-dpc donor mice into the brains of older hosts. Because the transplanted neurons underwent cell death at a time consistent with the age of the donor, rather than of the host, the authors concluded that interneuron cell death is determined by the intrinsic maturational state of the neurons (Southwell et al., 2012). Genetic and molecular regulators that could underlie such a developmental timing program have been well characterized in invertebrates (Grosskortenhaus et al., 2005; Moss, 2007), but are less studied in vertebrates. Our current data support the existence of an intrinsic developmental program, but one that can be accelerated by an advanced birth.

We also found effects of birth timing on overall magnitude of cell death in the neonatal brain, with an early birth decreasing cell death in the NAcc (but not CA1or) and a late birth increasing cell death in the CA1or (but not the NAcc). Because these effects were in opposite directions and in both cases regionally-specific, a generalized pathologic effect of advanced or delayed birth (within the normal range for the species) is unlikely to explain these results. Together with birth timing, other extrinsic factors also modulate cell death. For example, delivery mode (Castillo-Ruiz et al., 2018b) and the exposure to a microbiota at birth (Castillo-Ruiz et al., 2018a) affect the magnitude of cell death in the neonatal mouse brain. Moreover, effects are regionally specific in both cases, as in the current study. It is possible that these findings reflect regional variability in the sensitivity to factors that change at birth, such as hormones or cytokines. The hippocampus, for example, is especially sensitive to glucocorticoids (Sapolsky, 1996), so if the stress response following a delayed birth is greater than that of an on-time birth (as suggested by Rahman et al., 2017), hippocampal cell death might be affected.

Obstetric practices across the world now routinely alter human birth timing, usually to advance it. Elective cesarean-sections are, by definition, scheduled prior to 40 weeks gestation (Gibbons et al., 2010), and account for >15% of the births in the US and other countries (Betrán et al., 2016). Similarly, elective labor induction is common prior to full-term and advances birth timing (Salemi et al., 2016). The ability of birth to advance cell death could be an adaptation that reflects the importance of cell pruning for the maturation of brain circuits. However, if other neurodevelopmental events do not follow suit, as suggested by our measurements of total brain growth, cell death timing may be uncoupled from other aspects of brain development. This could be problematic, because tight temporal control is a basic feature of neural development (Durand and Raff, 2000; Rougvie, 2005; Moss, 2007), and disruptions can lead to developmental defects (Bateman and McNeill, 2004). Changes in the overall magnitude of neonatal cell death may lead to long-term changes in neuron number, as shown for effects of birth mode in mice (Castillo-Ruiz et al., 2018b). Because naturally-occurring cell death in the human brain is ongoing at birth (Lossi et al., 1998; Abrahám et al., 2001; Lavezzi et al., 2006; Abitz et al., 2007), it is possible that some of the reported effects of birth timing on child development (El Marroun et al., 2012; Abel et al., 2017; Heuvelman et al., 2018) are related to alterations in cell death, or a dissociation of cell death from other neurodevelopmental events

## Synthesis

Reviewing Editor: Margaret McCarthy, Univ of Maryland School of Medicine Decisions are customarily a result of the Reviewing Editor and the peer reviewers coming together and discussing their recommendations until a consensus is reached. When revisions are invited, a fact-based synthesis statement explaining their decision and outlining what is needed to prepare a revision will be listed below. The following reviewer(s) agreed to reveal their identity: Christine Wagner.

In this manuscript the authors describe the temporal pattern and magnitude of cell death in the CA1 oriens layer of the hippocampus and the NAcc shell in mice that were born on time, or with experimentally advanced or delayed birth. Their hypothesis is that cell death patterns and magnitude may be controlled by either the birth process or by an intrinsic developmental program. The authors find evidence to support both possibilities: advancing birth with RU-486 administration triggers an earlier onset of cell death in these two regions, with a reduction in the magnitude of cell death in the NAcc, while delaying birth by administering progesterone does not alter the timing of cell death but alters the magnitude of cell death in the CA1or.

The results of this study are interesting, and overall the methods seem appropriate for addressing the hypothesis. While the study is mainly descriptive and limited in scope, the technical limitations are appropriately discussed in the manuscript and the results represent an advance in our understanding of how birth can affect perinatal cell death. However, some major methodological and minor organizational concerns remain that should be addressed before publication.

This is a very well-written manuscript that addresses a highly novel, and developmentally important, question. The experiments are carefully executed and analyzed and the results are very clear with significant implications for our understanding of neurodevelopment and the timing of birth in humans. Overall, this paper is outstanding and only a few comments and suggestions arise.

1) Methodology: Cell death was assessed by quantifying AC3-labeled cells in the regions of interest. Insufficient detail is provided for the methodology in the sections “Quantification of AC3 labeling” and “Brain regions and total forebrain volume” to accurately assess how the quantification was carried out. For instance, it is unclear why quantification performed using different software for pups (Aperio Image Scope) vs fetuses (Stereoinvestigator)

2) Despite expression of density in mm3, it is unclear from the information provided in the methods whether the quantification used in this study can be considered ‘stereological’(i.e. three-dimensional interpretation of two-dimensional cross sections of materials or tissues) or unbiased in its design. Information should be provided on number of sections counted from, distance between sections, and how volume of regions was determined (e.g. Cavalieri method). This is essential for comparable density measurements across the experiment.

3) How were cells sampled within contours? Were all cells counted or were they randomly sampled? If counting was indeed stereological, what were the coefficient of error parameters for each region?

4) For “Brain regions and total forebrain volume”, it appears region volumes were estimated using only 4 consecutive 40um sections. This is unlikely to cover the entire region(s) of interest, and may introduce a confound in the density measurement by sampling different subsections of the region of interest. More information about how the analysis was designed, including citations of other work using this type of analysis, would be helpful in clarifying whether the quantification is appropriately unbiased.

5) Methods: A more thorough explanation of the statistical approach used to examine cell death “pattern matching” should be included (e.g., why Z-scores?). As only two data points (pcd 19 and 20) were common to both groups and were the only time points used in the analysis, it is unclear how differences in the timing or pattern were determined with only two time points.

Information on the software used to perform statistical analysis should be included

6) Figures: The manuscript is short and contains only three main figures. It also includes 4 extended data figures, each of which consists of only 1-2 graphs. Reorganizing the figures to include all data in the main manuscript will improve readability.

7) There are discrepancies in the figure legends and references to figures in the text. Extended data: Legends are included for figures 1-1, 2-1, 3-1, 4-1; Extended data figures 1-1, 2-1, 2-2, and 3-1 are mentioned in the text and main figure legends.

8) Abstract: It might be beneficial to add to the Abstract that activated caspase-3 was used as a marker of cell death in this study.

9) Methods: The time of day at which injections of RU486 or progesterone were administered should be added. Are there data on how many hours post-RU486 birth occurred?

10) Methods: There is no information provided on the timing or birth outcomes in progesterone treated mothers. Did all mothers administered progesterone give birth normally on day 20? Was the timing of birth more variable than in the RU486 treated mothers?

11) Results: The question of whether RU486 or progesterone alone is sufficient to alter cell death (independent from the timing of birth) is an important one. The complexities of administering these agents and assessing cell death within a time window prior to effects on birth timing is appreciated. However, while 3h post-injection is sufficient for the drug to access the fetus (as cited by the authors) and is sufficient to exert any rapid membrane progestin receptor effects, the slower transcriptional effects of nuclear progesterone receptor action on cell death are unlikely to have occurred within 3 hours.

12) The Discussion is very well-written and highly informative.
