# Supplementary figures and images for: Does Birth Trigger Cell Death in the Developing Brain?
Source: eNeuro. 2020 Feb 10;7(1):ENEURO.0517-19.2020. doi: 10.1523/ENEURO.0517-19.2020 (PMC7031855; doi:10.1523/ENEURO.0517-19.2020)

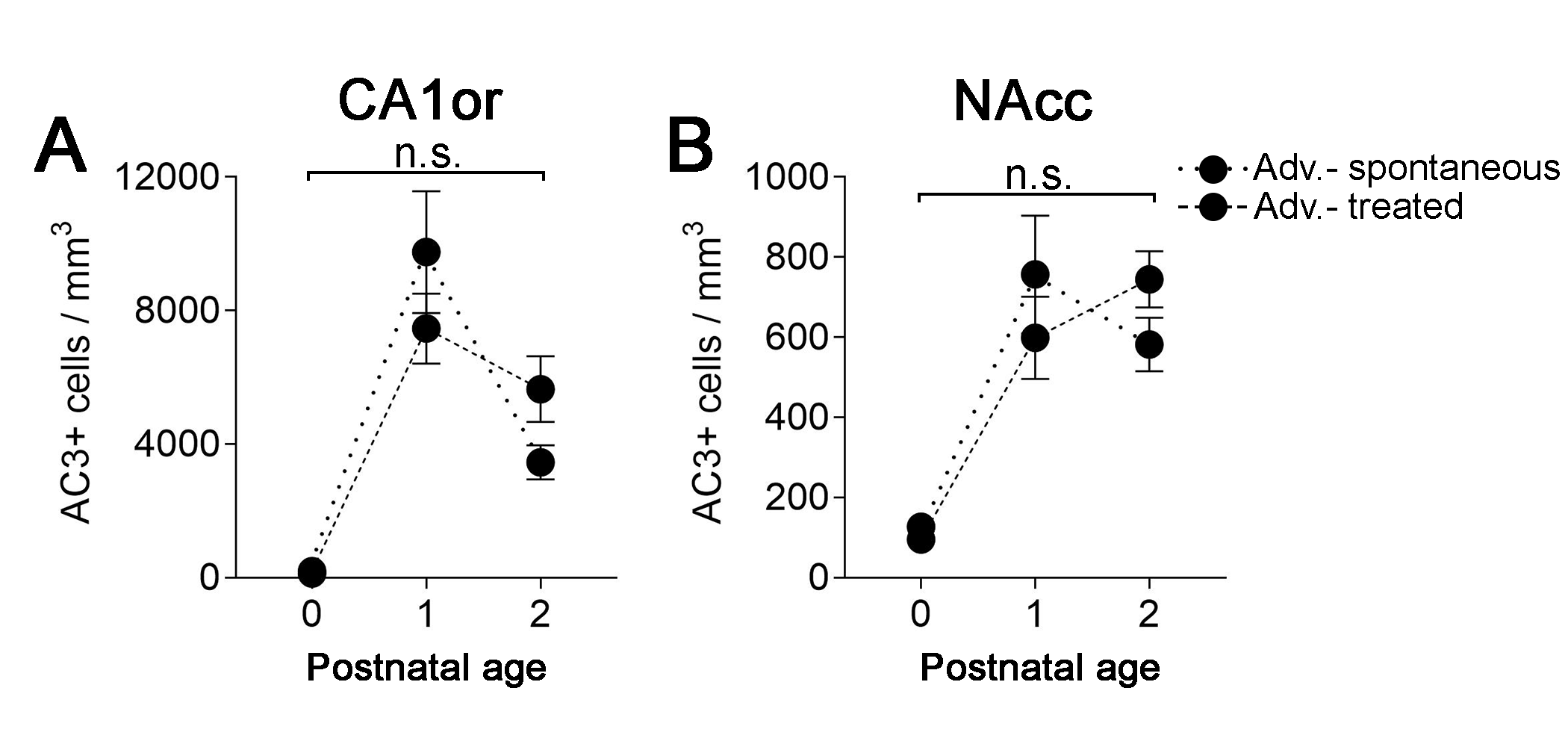

Supplement: Extended Data Figure 1-1 — Cell death patterns are similar between mice with spontaneous advanced birth and those with advanced birth after RU-486 treatment. AC3 cell density in the CA1or (A) and NAcc (B) are depicted for mice spontaneously delivered at 18 dpc (dotted lines) versus those delivered at 18 dpc after RU-486 treatment of the dam (hashed lines). In both cases, cell death is very low on P0 and increases significantly on P1, with no significant differences between groups; n.s., non-significant; N = 8–12 mice per group. Means ± SEM are plotted. Download Figure 1-1, TIF file. [file enu-eN-NWR-0517-19-s02.tif]
